# Associations between Relational Pronoun Usage and the Quality of Early Family Interactions

**DOI:** 10.3389/fpsyg.2016.01719

**Published:** 2016-11-01

**Authors:** Sarah Galdiolo, Isabelle Roskam, Lesley L. Verhofstadt, Jan De Mol, Laura Dewinne, Sylvain Vandaudenard

**Affiliations:** ^1^Psychological Sciences Research Institute, Université catholique de LouvainLouvain-la-Neuve, Belgium; ^2^Department of Experimental Clinical and Health Psychology Universiteit GentGhent, Belgium

**Keywords:** family alliance, we-ness, separateness, early family interactions, pronoun usage

## Abstract

Our study examined the relationships of relational pronouns used in parental conversation to the quality of early family interactions, as indexed by Family Alliance (FA). We hypothesized that more positive family interactions were associated with the use of more we-pronouns (e.g., we, us, our; *we-ness*) and fewer I- and you-pronouns (e.g., I, me, you, your; *separateness*) by both mothers and fathers. Our statistical model using a multilevel modeling framework and two levels of analysis (i.e., a couple level and an individual level) was tested on 47 non-referred families (*n* = 31 primiparous families; child’s age, *M* = 15.75 months, *SD* = 2.73) with we-ness and separateness as outcomes and FA functions as between-dyads variables. Analyses revealed that we-ness within the parental couple was only positively associated with family affect sharing while separateness was negatively associated with different FA functions (e.g., communication mistakes). Our main finding suggested that the kinds of personal pronouns used by parental couples when discussing children’s education would be associated to the emotional quality of the family interactions.

## Introduction

Mother: “Concerning our son’s education, we try to teach him values.”

Father: “Our values.”

Mother: “Yes, our values—and to do our best”.

These comments were made during a parental conversation on family, childrearing and education in our study. The parents used *we-*pronouns to refer to important aspects of family life, whereas other parents might have concentrated more on their personal opinions and used *you-* and *me-*pronouns. Was such pronoun use simply fortuitous, or was it related to the degree of early family engagement and coordination? The aim of the current paper was to examine the association between pronouns used during parental conversations and quality of early family interactions.

### We-ness Versus Separateness

The constructs of we-ness and separateness have long been studied in couples research (Singer et al., 2015). We-ness has been shown to reflect a schema of interdependence, shared responsibility, and partnership, while separateness would reflect one of independence and a focus on the individual spouses rather the couple as a unit. According to the investment model of interdependence, increases in relationship commitment should lead to a sense of we-ness and consequently to a common couple identity in which “mental representations of the couple became more prominent” (Agnew et al., 1998). Researchers have employed natural language usage as an implicit measure of we-ness and separateness. Psycholinguistic research (Pennebaker and Stone, 2003) has shown that the words we speak may reveal components of our psychological state such as emotional feelings, social identity, and cognitive style. Current research that has taken pronoun usage into account in the context of couple relationships was encouraging. First-person plural pronouns (e.g., *we, our, us)* seemed to reflect shared experience while first-person (e.g., *I, my, me*), while second-person singular (e.g., *you, your*) pronouns would reflect individuated experiences. Individuals who preferentially employed we-pronouns rather than you- and me-pronouns perceived their relationships as more intimate and of higher quality (Fitzsimons and Kay, 2004). Seider et al. (2009) found evidence that pronoun use was associated with the emotional quality of couple interactions and to couple satisfaction. Specifically, they reported that we-ness language correlated positively to interactions characterized by low levels of negative emotional behavior and high levels of positive emotional behavior. The opposite pattern of results was found for separateness. Finally, Seider et al. (2009) reported that older couples showed greater levels of we-ness and a greater sense of shared identity because of the greater number of shared experiences.

### Early Family Interactions as a Context of Interdependence

Until now, the constructs of we-ness and separateness as assessed by natural language and pronoun usage have never been studied in the family context, even though early family interactions, especially, have been seen as an example of a context of interdependence which involves a partial transformation of identity (Agnew et al., 1998). Childbirth implies the arrival of another individual in the family and in self- and relational awareness (Aron et al., 1992). A model of early family interactions has been developed called “Family Alliance” (FA), which concerned “the degree of early family engagement and cooperation in everyday activity involving triadic family interactions (i.e., mother-father-child),” such as playing together or having a meal (Favez et al., 2010). The aim of the current study was to analyze whether we-ness and separateness language were associated with FA.

### Current Study and Hypotheses

In the current study, we examined pronoun usage during a minimally structured parental conversation about the parents’ child’s education. This study was thus the first to include a measure of we-ness and separateness arising from conversational text analysis and the observational assessment of early family interactions, all obtained during actual family interactions. According to the investment model of interdependence, and because family is a context of interdependence, we hypothesized that more positive family interactions would be associated with the use of more we-pronouns and fewer I- and you-pronouns by both mothers and fathers. Like Fitzsimons and Kay (2004), we expected that parents who preferentially used we-pronouns as opposed to you- and me-pronouns would show a higher degree of family engagement and coordination. Secondly, like Seider et al. (2009) we expected that, because of shared experiences in couple and family life, long-term couples and multiparous parental couples would use more we-pronouns and fewer separateness-pronouns. Hypotheses were tested in a multilevel modeling framework.

## Method

### Sample

Data were collected within a sample of 47 non-referred French-speaking heterosexual families (*n* = 31 primiparous families). The children were 30 girls and 17 boys (Age, *M* = 15.75 months, *SD* = 2.73). Parents were aged from 23 to 43 years old (*M* = 28.87, *SD* = 3.63) and were paired as couples (Relationshipduration, *M* = 7.63, *SD* = 3.50). Participants were recruited with the help of seven gynecologists from hospitals who gave information about the current study to their patients verbally and by means of a flyer.

### Procedure

Data were collected using the Lausanne Trilogue Play procedure (LTP; Fivaz-Depeursinge and Corboz-Warnery, 1999) which was a semi-standardized observation play situation including mother, father, and baby together. Experimenters asked parents to sit in front and on each side of their child so that the three were arranged in a triangle. Technical equipment consisted of two cameras, one recording the parents and one the child. Researchers gave the following guidelines: “We’ll ask you to play together as a family in four separate situations. In the first, one of you plays with the child and the other one is simply present. In the second, you reverse the roles. In the third, the three of you will play together. In the last part, you (i.e., parents) will talk a while together about how you raise your child, and it will be the child’s turn to be simply present.” The research team randomly decided who began the game in order to counterbalance any possible order effect between the mother and father. The partners were allowed to interact for as long as they considered necessary, without any time limit. The mean duration of the LTP in this study was 14 min, 34 s (*SD* = 4.47), instructions included. Before participating in the LTP, parents were asked to separately complete a brief questionnaire on relationship satisfaction. For ethical reasons, this study was registered with the Commission for the Protection of Privacy in Belgium. Parents signed an informed consent and were assured that the collected data would remain confidential.

### Measures

#### Family Alliance

Family Alliance was evaluated with the Family Alliance Assessment Scale (FAAS; Favez et al., 2010), which included 11 scales operationalizing the five functions of FA, as described in **Table 1**. These functions represent interactional family patterns and were needed for establishing a successful interaction. Each scale allowed an evaluation of the family interaction according to an ordinal scoring system in three points: *appropriate* (2 points), *moderate* (1 point), and *inappropriate* (0 point). We used FAAS to analyze family interactions during the LTP. During the last part of the LTP, we asked the parents to talk to one another about the way they raised their child and to give their opinions about education-related questions (e.g., setting limits, rewarding, showing love and affection; Duration, *M* = 3.35, *SD* = 1.52). The scores of the different scales were added to obtain the score of each function. The higher the scores of the functions were, the more positive was the FA. Previous evidence for the reliability and validity of the FAAS functions can be found in Favez et al. (2010).

**Table 1:** The Family Alliance Assessment Scale (FAAS) scales and functions (Favez et al., 2010).

Functions	Scales	Description of appropriate criteria
Participation	Postures and gazes	The non-verbal cues indicate readiness and willingness to interact with one another.
	Inclusion of partners	All family members are included in the interaction.
Organization	Role implication	Each partner performs his or her role during the play.
	Structure	The expected interactive structure is respected.
Focalization	Co-construction	All partners share the topic of the game.
	Parental scaffolding	Stimulation is adapted to the child’s age and developmental stage.
Affect sharing	Family warmth	Affects are mainly positive.
	Validation	Partners adjust to each other’s emotional states.
	Authenticity	Affects are congruent with the situation.
Timing	Interactive mistakes during activities	There are few communication mistakes, and these are rapidly corrected
	Interactive mistakes during the transitions	When a change in activity occurs, the interaction is organized in a smooth manner.


One certified coder (i.e., trained in LTP coding) coded all the videos. Two additional coders each coded half of the videos (videotaped interactions were randomly assigned to one of two coders), so that all the videos were double-coded to test inter-rater reliability. Inter-rater reliability (i.e., intra-class coefficient, ICC) ranged from 0.61 to 0.90, with an average of 0.80, all correlations being significant to at least *p* < 0.05. High internal consistency across 11 scales of FA (α = 0.92) was also observed. In our sample, a five-factor solution relative to the five functions emerged, explaining 82.60% of the variance. αs ranged from 0.63 to 0.80 and ICC was 0.87.

#### We-ness and Separateness

Two trained research assistants coded the we-ness and the separateness data in accordance with the coding procedure developed by Seider et al. (2009). The first step consisted in the verbatim transcription of the videotaped part 4 of the LTP and of identifying pronouns used by the parents when talking about child’s education. Secondly, each pronoun was classified in one of three categories: (a) me-pronouns referring to the self, (b) you-pronouns referring to the partner, and (c) we-pronouns referring to the parental couple. Similar to Seider et al. (2009), the verbal context of participants’ pronouns was considered as well, given its influence on the meaning of a particular pronoun. For example, “pronouns used as part of an idiomatic expression” used to fill a pause in conversation (e.g., “*I* don’t know…”) were not classified in one of the categories above (for more details on the contextual analysis, see Seider et al., 2009). Finally, the number of we-pronouns of each mother and father was divided by the total number of words spoken by the mother and father, respectively (i.e., we-ness). The number of me-pronouns and you-pronouns for each mother and father were summed and then divided by the total number of words spoken by the mother and father, respectively (i.e., separateness; Hinnekens et al., 2016). Scores potentially ranged from 0 to 1. This procedure resulted in two pronoun variables for each parent, namely the we-ness and the separateness constructs. Reliability for this coding in the current study was very high (Cohen’s kappa = 0.98).

#### Relationship Satisfaction

The Kansas Marital Satisfaction Scale (KMSS; Schumm et al., 1983) was a 3-item measure designed to assess relationship satisfaction with a 7-point Likert-type scale (1 = *Extremely dissatisfied* and 7 = *Extremely satisfied*). High internal consistency (α = 0.94) was observed. Each partner’s score was separately entered in the analysis.

## Results

### Statistical Analysis

Data were analyzed using hierarchical linear modeling (HLM 6.08). Our model included two levels of analysis, i.e., a couple-level (level 2 data) and an individual-level (level 1 data) with we-ness and separateness scores separately as the outcomes. Three kinds of predictors were included, i.e., between-dyads, within-dyads, and mixed variables. Between-dyads variables (i.e., variables with the same score for both partners in a couple, but different from couple to couple, i.e., FA functions, couple’s relationship duration, and primiparity/multiparity) were introduced at the level 2 data (i.e., couple-level). Within-dyads variables (i.e., different within a single couple but similar between couples, i.e., gender) and mixed variables (i.e., variation both within the couple and between couples, i.e., relationship satisfaction and age) were introduced at the level 1 data (i.e., individual-level). Age, gender, and relationship satisfaction were used in the statistical model as control variables. Note that because of close correlations between the different FA functions, with 0.35 < *r* < 0.81, *p* < 0.001, we used the residuals of the FA functions as between-dyads variables (for more details, see **Table 1**).

### HLM Model

Preliminary analyses indicated that the means, standard deviations, and ranges for the we-ness and separateness measures were 0.04 for both constructs (*SD* = 0.03 for we-ness and separateness; Range: 0.00–0.15 and 0.00–0.18 for we-ness and separateness, respectively). Significant negative correlations were found between the two types of pronouns both for mothers, *r* = -0.42, *p* = 0.003, and fathers, *r* = -0.57, *p* = 0.000. Examining the size of the correlation coefficients suggested that there was 18 and 32% shared variance between we-ness and separateness pronouns for mothers and fathers, respectively. The percentage of shared variance led us to treat the constructs of interest separately.

Two conditional models were tested with we-ness and separateness as outcomes, respectively. These models allowed us to test our main hypothesis, which questioned whether more positive family interactions were associated with the use of more we-pronouns and fewer I- and you-pronouns by both mothers and fathers. **Table 2** depicts the results of the conditional models of we-ness and separateness with the FA functions as predictors. For we-ness, significant positive effect of affect sharing (β = 0.012, *p* = 0.009) was found: The more the family shared warmth and positive affect, the more we-ness pronouns were used during parental conversation. For separateness, significant negative effects of participation (β = -0.020, *p* = 0.007), organization (β = -0.021, *p* = 0.020), affect sharing (β = -0.028, *p* = 0.008), and timing (β = -0.022, *p* = 0.013) were found. A gender-effect (β = -0.010, *p* = 0.040) was observed: Within the couple, fathers tended to use more separateness pronouns than mothers. Finally, except for the FA functions, the other variables did not play any important role in the prediction.

**Table 2:** Multilevel conditional models of we-ness and separateness with Family Alliance (FA) functions.

Parameter	We-ness		Separateness	
		
	Estimate	*SE*	Estimate	*SE*
**Fixed**				
Intercept	0.017^∗∗∗^	0.002	0.046^∗∗∗^	0.004
**Level 2**				
Between-dyads variables				
Participation	0.006	0.003	-0.020^∗∗^	0.006
Organization	0.003	0.004	-0.021^∗^	0.008
Focalization	0.009	0.005	-0.013	0.009
Affect sharing	0.012^∗∗^	0.004	-0.028^∗∗^	0.009
Timing	0.007	0.004	-0.022^∗∗^	0.008
Primiparity/multiparity	0.003	0.002	-0.008	0.005
Duration of the couple	0.000	0.001	-0.001	0.001
**Level 1**				
Within-dyad variable				
Gender	0.000	0.001	-0.010^∗^	0.004
Mixed variables				
Relationship satisfaction	-0.001	0.003	0.009	0.007
Age	0.000	0.000	-0.003	0.002
Deviance	-211.179		-130.608	


*Coefficients are standardized. *N* = 47. ^∗^*p* < 0.05; ^∗∗^*p* < 0.01; ^∗∗∗^*p* < 0.001.*

## Discussion

The aim of this research was to determine whether personal pronouns utilized during a parental conversation were related to early family interactions, indexed here by FA. According to the investment model of interdependence, we expected that more positive family interactions would be associated with the use of more we-pronouns and fewer I- and you-pronouns by both mothers and fathers. First of all, our findings provided substantial evidence that we-ness and separateness were two distinguishable constructs and, *de facto*, did not constitute a continuum because of (a) the weak percentage of shared variance between we-ness and separateness and (b) their associations with different FA functions.

Secondly, we-ness and separateness were found to be, respectively, positively and negatively related to affect sharing during family interactions. When family members shared (positive) emotions, validated each other’s emotions, and showed affect congruence, more use of we-pronouns and less use of I- and you-pronouns were observed. We-ness may be considered a schema of interdependence in a context of interdependence, as family relationships were considered so-called close relationships, defined as relationships with a “strong, frequent, and diverse interdependence that lasts over a considerable period of time” (Kelley et al., 1983). Our findings suggested that a feeling of we-ness or interdependence in a family was an affective phenomenon and involved the recognition and validation of family members’ emotions (Reis et al., 2002; Reis, 2014). Previous research has already shown that we-ness and separateness, as measured by pronoun usage, were related to an individual’s (Rude et al., 2004) and couple’s (Seider et al., 2009; Gildersleeve, 2015) emotional states, but this has not been demonstrated in relation to family affect sharing. Our results suggested that the kinds of personal pronouns used by parental couples when discussing children’s education were associated with the emotional quality of family interactions. Such results may be particularly relevant from a clinical perspective. The association between language and emotional states would imply that language reflected the emotional states of family interactions, but also that attention to pronoun use could help family members to conceive themselves as in closer partnerships. The narrative approach (Fitzsimons and Kay, 2004; Freedman and Combs, 2008) has already underlined the importance of language use in shaping perceptions of reality and especially the fact that manipulating pronoun usage can lead individuals to perceive their own relationships as higher in quality. Narrative researchers (Fitzsimons and Kay, 2004) have shown that using the we-pronoun led to increased perceptions of the closeness and quality friendships and interactions. The influence of pronoun use on closeness was shown to be partially mediated by perceptions that close individuals were similar and shared common values. Consequently, family clinicians could use words such as “all of you” for inducing a sense of interdependence, positively influencing affect sharing during family sessions.

Thirdly, while we-ness was only related to affect sharing, separateness words, which reflected a concentration on the individuals and a schema of independence, were negatively correlated to many FA functions, i.e., participation, organization, affect sharing, and timing. Early family interactions characterized by the exclusion of a family member, interferences, a lack of affect congruence, and communication mistakes were related to the use of more I- and you-pronouns during parental conversations. Previous research and clinical practice has indicated the importance of the autonomy of each individual in his or her relationships (Benson et al., 2013). However, our results suggested the importance of paying attention to such separateness language during parental conversations concerning a child’s education and upbringing.

Fourthly, the second hypothesis was not confirmed: Long-term and multiparous couples did not use more we-pronouns and fewer separateness-pronouns than newer and primiparous couples. Previous research (Seider et al., 2009) has shown that older couples used more we-pronouns during couple conversation than younger couples, due to having more shared experience. However, this was not evident in early family interactions. Our results suggested that couple duration and family experiences would not lead to a greater sense of we-ness. This could be due to the fact that the span used in this study and the ranges of age and couple duration were small compared to previous studies comparing younger and older adults.

The current research extended prior studies on we-ness and separateness in many ways: (a) using schemas of we-ness and separateness derived from pronouns use during natural parental conversation, (b) employing text analysis methodology, and (c) studying associations between parental conversation and family interactions. Limitations included the absence of a study of actor-partner interaction effects and the weak sample size.

## Author Contributions

SG collected the data, made the statistical analyses and wrote the paper. IR helped for the analyses and the writing of the paper. LV and JD are specialized in family and couple interactions and gave a lot of recommandations. The idea of analyzing we-ness and separateness came from LV. LD and SV contributed to the literature section.

## Conflict of Interest Statement

The authors declare that the research was conducted in the absence of any commercial or financial relationships that could be construed as a potential conflict of interest.
